# Serum Neurofilament Light Chain: A Marker of Nervous System Damage in Myopathies

**DOI:** 10.3389/fnins.2021.791670

**Published:** 2021-12-17

**Authors:** Annika Saak, Pascal Benkert, Katja Akgün, Eline Willemse, Jens Kuhle, Tjalf Ziemssen, Sandra Jackson, Jochen Schaefer

**Affiliations:** ^1^Department of Neurology, University Hospital Dresden, Dresden, Germany; ^2^Department Klinische Forschung, University Hospital Basel, Basel, Switzerland; ^3^Department of Neurology, University Hospital Basel, Basel, Switzerland

**Keywords:** myopathy, serum neurofilament, nervous system involvement, mitochondriopathies, myotonic dystrophies (DM1 and DM2), facio scapulo humeral dystrophy

## Abstract

**Purpose:** Neurofilament light chain in serum (sNfL) has been suggested as a biomarker for the assessment of neuroaxonal damage. Since NfL are not expressed in muscle, elevated sNfL in patients with primary myopathies suggest additional nervous system involvement. To verify this hypothesis, we measured sNfL in a series of patients with myopathies.

**Methods:** sNfL were determined in 62 patients with molecular proven primary myopathies in whom some nervous system involvement may be predicted: myotonic dystrophy type I and II (DM I, II) and mitochondrial disease. In addition, sNfL were measured in 8 patients with facioscapulohumeral muscular dystrophy (FSHD) and in a disease control group caused by genetic defects exclusively expressed in muscle.

**Results:** sNfL values were significantly elevated in the DM I, the DM II and the mitochondrial group, with FSHD patients showing the lowest sNfL elevations. sNfL levels in the disease control group were not different from the healthy controls. A significant correlation between repeat length and sNfL levels was found in the DM I patients, but not in the DM II patients. Mitochondrial patients with encephalopathy showed significantly higher sNfL concentrations compared to patients with only muscular symptoms.

**Conclusion:** sNfL levels are elevated in myopathies with, based on the underlying molecular defect or clinical features, established nervous system involvement, i.e., myotonic dystrophies and mitochondrial disorders. sNfL were also raised in FSHD, where involvement of the nervous system is not usually clinically apparent. Thus, sNfL concentrations may serve as a biomarker for additional neuronal damage in primary myopathies.

## Introduction

Primary myopathies are a diverse group of genetic and acquired conditions, which may present with a plethora of clinical symptoms, and it is becoming increasingly evident that many hereditary primary myopathies can be ascribed to underlying multisystem disorders. MRI studies have uncovered central nervous system (CNS) involvement, with evidence of cerebral atrophy, gray and white matter lesions and ventricular enlargement in a number of muscle diseases (Angelini and Pinzan, 2019). These diseases include certain muscular dystrophies (e.g., Duchenne), the dystrophic myotonias and mitochondrial myopathies.

The myotonic disorders are classified into dystrophic (myotonic dystrophy Type I/DM1 and myotonic dystrophy Type II/DM 2/PROMM) and non-dystrophic myotonias. The underlying genetic defect in the myotonic dystrophies affects transcription and translation of multiple cellular genes, thus also causing extramuscular pathology, with cardiac, endocrinological and CNS-symptoms.

The mitochondrial diseases represent the archetype of multisystem disorders because of the ubiquitous occurrence of mitochondria, but they also display a high degree of heterogeneity due to the involvement of either the nuclear or mitochondrial genome and, in the case of mitochondrial DNA mutations, frequent disparity in the level of mutations between the different tissues. Not surprisingly, because of their high energy requirements, both the peripheral and the central nervous system are frequently involved in mitochondrial diseases. Our cohort included patients with proven defects of nuclear genes (*POLG*, encoding the mitochondrial polymerase gamma and *DNAJC30*, encoding DnaJ heat shock protein family (Hsp40) member C30) and of mtDNA encoded mitochondrial genes (*MT-ATP6*, encoding ATPase 6, *MT-TL1*, encoding tRNA Leucine 1, *MT-TK* encoding tRNA Lys, *MT-T1* encoding tRNA Ile) and patients with a single, large scale deletion in mtDNA.

Clinically evident CNS involvement is rare in facioscapulohumeral muscular dystrophy (FSHD), but detailed neurophysiological testing has shown subclinical involvement in a number of cases (Stübgen, 2007).

Until now, the assessment of CNS involvement in myopathies has mainly been based upon clinical parameters such as neuropsychological testing or cerebral imaging by MRI. Therefore, there is a need for reliable and easily accessible biomarkers for the detection of relevant nervous system damage, possibly even at a pre-symptomatic stage.

Neurofilaments constitute an important part of the neuronal cytoskeleton and are relevant for axonal growth and transport. Elevated levels of serum neurofilament light chain have been shown to specifically reflect neuroaxonal damage in a variety of neurodegenerative conditions such as amyotrophic lateral sclerosis (Steinacker et al., 2016), Alzheimer’s dementia (Mattsson et al., 2017) and multiple sclerosis (Kuhle et al., 2017). Plasma or serum NfL have also been measured in peripheral nervous system disorders, including Charcot-Marie-Tooth disease (Sandelius et al., 2018), the acquired polyneuropathies (Mariotto et al., 2018; Van Lieverloo et al., 2019) and Guillain-Barré syndrome (Martín-Aguilar et al., 2021). In all cases, increased NfL levels were associated with greater disease activity and severity, thus monitoring of serum NfL levels may also be used to assess disease progression.

The aim of this study was to ascertain whether, using sNfL as a non-invasive, easily accessible biomarker, nervous system involvement can be demonstrated in patients with primary myopathies. We chose three multisystem disorders, DM1, DM2/PROMM, and mitochondrial disease, with frequent nervous system involvement and one myopathy without overt nervous system involvement (FSHD) to test this hypothesis. In addition, we measured sNfL concentrations in a group of disease controls, consisting of monogenic primary myopathies caused by pathogenic mutations in genes which are expressed in muscle tissue only. These comprise structural muscle proteins, channel proteins of the muscle membrane and proteins related to muscle energy metabolism.

## Methods

Clinical data and blood samples were prospectively collected in the Neuromuscular Outpatient Clinic of the University Hospital Dresden, Germany. Molecular genetic confirmation of the diagnosis was available for all patients. Patients with known acquired diseases or injuries of the CNS were excluded from the study. The NfL study was approved by the local Ethics committee (ID: EK394102018), conforms with World Medical Association Declaration of Helsinki and the patients or their authorized representatives consented to the use and storage of the biosamples and publication of their data.

All serum samples were frozen immediately and stored at −20^°^C. sNfL concentrations were determined using the single-molecule array (SIMOA) analysis as previously described (Akgün et al., 2019; Sutter et al., 2020). The mean intra-assay coefficient of variation of duplicates and the mean inter-assay coefficient of variation was<10%. The control group comprised 485 samples from healthy volunteers, which were collected in the University Hospital Basel as previously described (Sutter et al., 2020).

To account for the known positive correlation of NfL with age, age-corrected sNfL percentile values have been derived based on healthy controls using a Generalized Additive Model for Location, Scale and Shape (GAMLSS) as described previously (Sutter et al., 2020). The percentile value was then calculated for each data point. Unadjusted group differences in sNfL levels were visualized using boxplots and were assessed using the Wilcoxon rank sum test. To additionally account for the minor age differences between groups, a multivariable linear regression model with log (sNfL) as the dependent variable was built with group and age as predictors. Estimates were back-transformed (exponentiated) and therefore represent multiplicative effects on the geometric mean of sNfL. Correlation of sNfL with age and repeat length within a patient group were assessed using Pearson correlation coefficients. Differences between different groups of mitochondrial patients were assessed using the unpaired *t*-test (GraphPad Prism). All remaining analyses were done with the statistical Software R (version 4.1.0). We used the STARD reporting guidelines (Bossuyt et al., 2015).

## Results

Between December 2019 and September 2021, 62 patients with either DM1, DM 2/PROMM, mitochondrial disease or FSHD and 13 patients for the disease control group were recruited for the study. Clinical characteristics and demographics of the patient cohorts are shown in Table 1.

**TABLE 1:** Clinical characteristics and demographics of the patient cohorts.

A.					
**Patient group**	**sNfL [pg/ml]**	**Age/Gender**	**Co-morbidity/Neurological medication**	**Repeat expansion/Diagnosis (Mutation)**	**Tissue expression**
**DM I**					
1	**6**	32, f	Lamotrigine (myotonia)	150	
2	**11**	37, f	—-	n.a.	
3	**24.1**	52, m	—-	500	
4	**8.5**	39, m	Dissociative seizures/lamotrigine (myotonia)	250	
5	**11.5**	57, f	—-	220	
6	**11.2**	29, f	—-	400	
7	**55.8**	67, f	—-	220	
8	**10.7**	31, m	Lamotrigine (myotonia)	300	
9	**9.06**	55, f	—–	200	
**DM II**					
1	**11.3**	53, f	—-	2,000	
2	**9.8**	49, f	—-	n.a.	
3	**15.5**	71, f	—-	5000	
4	**21.4**	70, f	—-	n.a.	
5	**7.5**	33, f	—-	n.a.	
6	**11.5**	58, f	—-	7,000	
7	**8.7**	51, f	Depression/citalopram	4,000	
8	**15.6**	60, f	—-	n.a.	
9	**12.6**	53, f	Fibromyalgia	n.a.	
10	**12.8**	55, f	—-	n.a.	
11	**9.8**	38, m	—-	7,000	
12	**26.4**	64, m	—-	7,000	
13	**8.8**	51, m	Chronic pain/duloxetine, mirtazapine, pregabalin	4,500	
14	**4.7**	25, f	—-	3,500	
15	**3.6**	19, f	Migraine/—	3,000	
16	**12.1**	41, f	—-	4,000	
17	**9.3**	50, m	—-	6,500	
18	**4.2**	33, m	—-	1,500	
19	**8.0**	44, m	Epilepsy/valproate	n.a.	
20	**6.1**	41, f	Restless legs syndrome	4,000	
21	**4.6**	33, m	Essential tremor/venlafaxine	n.a.	
22	**17.8**	57, f	Restless legs syndrome, depression/levodopa	4,000	
**FSHD**					
1	**10.9**	65, m	—-		
2	**13.7**	62, m	—-		
3	**11.3**	48, f	—-		
4	**4.8**	37, m	—-		
5	**9.0**	56, m	—-		
6	**10.3**	59, m	—-		
7	**16.7**	49, f	—-		
8	**6.9**	19, m	—-		
**Disease controls**					
1	**11.6**	41, f	Depression/duloxetin; lamotrigine (myotonia)	Myotonia congenita Thomsen	Muscle only
2	**5.9**	29, f	Migraine	McArdle’s disease	Muscle only
3	**6.2**	17, f	Lamotrigine (myotonia)	Myotonia congenita Becker	Muscle only
4	**8.3**	62, m	Restless legs syndrome	RYR1: het. (p.V2280I)	Muscle only
5	**5.9**	36, m	—	Bethlem myopathy/COL6A3: het. (p.Gly2068fs)	Muscle only
6	**12.4**	42, m	—-	Myotonia congenita Becker	Muscle only
7	**4.8**	32, f	—-	Ocular myositis	Muscle only
8	**8.2**	60, m	—-	LGMDR1/CAPN3: het. (p.Cys442Tyr, c.1746-20C > G)	Muscle only
9	**7.3**	53, f	Gabapentin, amitriptyline (myotonia)	Myotonia congenita Becker	Muscle only
10	**11.4**	64, f	—-	MYH7: het. (p.Met1429del)	Muscle only
11	**6.6**	43, m	—-	MYH7: het. (p.Met1429del)	Muscle only
12	**9.1**	42, m	Depression/mirtazapine	Pompe	Muscle only
13	**6.5**	21, m	—-	CPT2: (p.Arg231Trp; p.Leu178_Ile186delinsPhe)	Muscle only

**B.**					

**Mitochondrial myopathy**	**sNfL [pg/ml]**	**Age/Gender**	**Non-myopathic symptoms**	**Diagnosis/Mutation**	**Tissue expression/Heteroplasmy level**

1	**10.7**	53, m	—-	CPEO/mtDNA—single deletion	Muscle
2	**11.3**	54, f	—-	CPEO/mtDNA- single deletion	Muscle, urine, (not blood)
3	**8.7**	61, f	—-	CPEO/mtDNA—single deletion	Muscle
4	**10.1**	47, f	—-	CPEO/mtDNA—single deletion (6.3 kB)	Muscle, urine, (not blood), (70% in muscle)
5	**14.5**	68, m	—-	CPEO/mtDNA—single deletion (4.5 kB)	Muscle, urine, not blood
6	**30.9**	62, m	Ataxia, cataract, visual impairment, SNHL	CPEO-plus/mtDNA—single deletion (4.5 kB)	Muscle, urine, (not blood)(60% in muscle)
7	**23.4**	20, f	Short stature	CPEO- plus/mtDNA—single deletion (7.8kB)	Muscle, urine, blood
8	**12.2**	42, f	Dysarthria	CPEO-plus/mtDNA—single deletion	Muscle, urine, (not blood)
9	**25.9**	43, m	SNHL, encephalomyopathy, short stature, cognitive impairment, ataxia	CPEO-plus/mtDNA—single deletion	Muscle, urine, blood
10	**17.4**	41, m	Optic atrophy (10 year-history)	LHON/DNAJC30: hom. (p.Y51C)	Nuclear encoded
11	**13.7**	59, m	Polyneuropathy	POLG: het. (p.R627Q; Q1236H in cis)	Nuclear encoded
12	**6.3**	34, f	—-(daughter of patient 11)	POLG: het. (p.R627Q; Q1236H in cis)	Nuclear encoded
13	**18.2**	63, f	SANDO, Parkinson, SNHL	CPEO-plus/mtDNA—multiple deletions; POLG: het., domin. (p.F961S)	Nuclear encoded
14	**12.7**	32, f	Short stature, polyneuropathy, dysarthria	MT-ATP6: m.9185T > C (p.L220P)	Blood (100%)
15	**33.8**	48, m	Ataxia, dysarthria, epilepsy, optic atrophy, cognitive impairment, polyneuropathy	MT-ATP6: m.9198delC (p.D224Efs*)	Urine, blood, fibroblasts (100%)
16	**30.8**	59, m	Myoclonus, epilepsy, ataxia, SNHL	MT-TK: m.8344A > G in tRNA-Lys	Blood (79%)
17	**11.9**	55, m	Ataxia, myoclonus, epilepsy, headache	MT-TK: m.8344A > G in tRNA-Lys	Urine (88%)
18	**1250**	48, f	Cerebral atrophy, stupor, stroke-like episodes, MRI: necrotizing encephalomyelopathy, onset age 16 years	Leigh/MT-T1: m.4290T > C in tRNA-Ile	Blood, muscle, fibroblasts (100%)
19	**66.3**	34, f	Dementia, neuropathy, ataxia, epilepsy, SNHL/anticonvulsants	MT-TL1: m.3243A > G in tRNA-Leu1	Urine (71%), blood (37%)
20	**70.4**	62, f	Stroke-like episode, only SNHL until age 62	MT-TL1: m.3243A > G in tRNA-Leu1	Urine (49%), blood (18%)
21	**14.5**	30, f	Mild SNHL, migraine	MT-TL1: m.3243A > G in tRNA-Leu1	Urine (49%), blood (25%)
22	**26.5**	57, f	SNHL, depression/citalopram. Mother of P21	MT-TL1: m.3243A > G in tRNA-Leu1	Urine (57%), blood (15%)
23	**33.2**	31, f	Migraine, rhabdomyolysis	MT-TL1: m.3243A > T in tRNA-Leu1	Urine, blood (low), muscle (80%)

*DM I, myotonic dystrophy type 1; DM II, myotonic dystrophy type 2; FSHD, facioscapulohumeral muscular dystrophy; sNfL, serum neurofilament light chain; RYR1, Ryanodine receptor 1; LGMDR1, Limb-girdle muscular dystrophy, recessive Type 1; MYH7, Myosin heavy chain 7; CPT2, Carnitine palmitoyltransferase 2.*

*CPEO, chronic progressive ophthalmoplegia; mtDNA, mitochondrial DNA; SNHL, sensorineural hearing loss; LHON, Lebers hereditary optic neuropathy; POLG, Polymerase gamma; MELAS, Mitochondrial encephalomyopathy, lactic acidosis, and stroke-like episodes. SANDO, sensory ataxia, neuropathy, dysarthria and ophthalmoplegia.*

Concomitant neurological diseases (depression, dissociative seizures, fibromyalgia syndrome, migraine, chronic/neuropathic pain, restless legs syndrome, essential tremor) in some patients with DM I/DM II and the medication taken by the patients are not, according to current knowledge, associated with structural neuronal damage and would therefore not affect sNfL levels. Several patients in the mitochondrial group showed, in addition to the myopathy, clinically apparent CNS-symptoms (Table 1). In these patients with mitochondrial myopathy, the CNS-symptoms are a feature of the primary mitochondrial disorder and therefore clinically confirm neuronal damage underlying the sNfL elevation.

**FIGURE 1:**
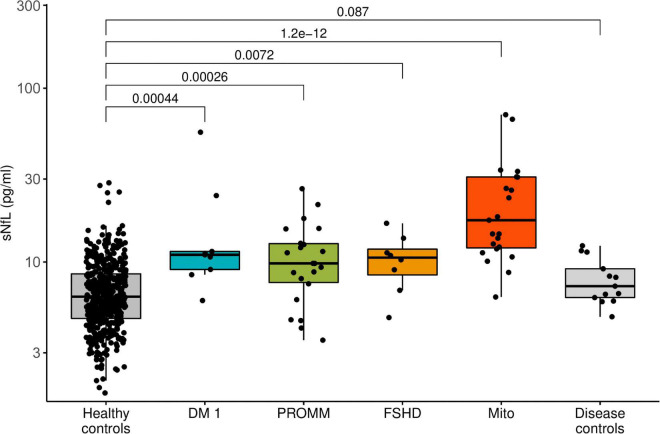
Boxplot diagram of serum neurofilament light chain (sNfL) concentrations, comparing the disease groups against healthy control samples. Boxes represent median and interquartile range (IQR) and whiskers the extreme value within 1.5 × IQR above and below the median. The *p*-values listed were determined using a Wilcoxon rank-sum test.

All disease groups showed significantly higher median serum NfL levels than the healthy controls, whilst the smallest elevation of sNfL concentrations was found in the FSHD group (Figure 1). After adjustment for age, serum NfL was on average 2.1 times higher (CI: 1.6;2.7, *p*-value: < 0.0001) in DM I, compared to levels in healthy controls, 1.4 times (CI: 1.2;1.7, *p*-value: < 0.0001) in DM 2/PROMM, 1.4 times (CI: 1.1;1.9, *p*-value: 0.0227) in FSHD and 3.2 times (CI: 2.7;3.8, *p*-value: < 0.0001) in the Mito-patient cohort.

**FIGURE 2:**
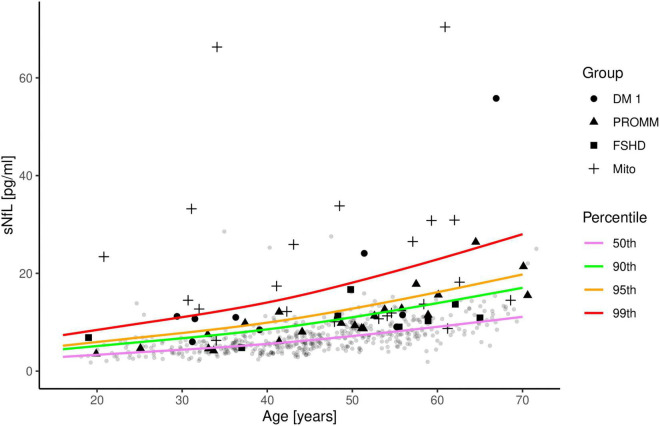
Serum neurofilament light chain (sNfL) concentrations in the four disease groups are shown in comparison to 485 control samples. The distribution of sNfL is shown as a function of age and expressed as percentile curves (coloured lines), based on the control samples (gray circles). Patient 18 from the mitochondrial group is not included in this figure as the sNfL value is outside the range of the axes.

Compared to the controls, serum NfL levels were above the 90th percentile in 6/9 (66%) patients with DM I and in 9/22 (41%) patients with PROMM (Figure 2). In the FSHD group, only 3/8 (38%) patients were above the 90th percentile. The mitochondrial patients had the highest NfL levels of all groups, and 16/23 (70%) patients had values above the 90th percentile (Figure 2).

Within each group, only the DM I and DM 2/PROMM patients showed a significant correlation between age and sNfL levels (*r* = 0.69, *p* = 0.04 and *r* = 0.83, *p* < 0.0001, respectively) (Supplementary Figures 1A,B), whilst no significant correlation was seen within the other groups (Supplementary Figures 1C–E).

No significant correlation was found between sNFL levels and repeat length in the DM2/PROMM patients (*r* = 0.5; *p* = 0.07), but there was a significant correlation in the DM I patients (*r* = 0.86; *p* = 0.014). P7 was omitted from the regression analysis as an outlier value, because the clinical symptoms (gait ataxia out of proportion to the myopathy), noted at the last examination when the sample was obtained, suggested additional neuronal pathology.

The mitochondrial cohort is a more heterogeneous group than the other groups, in terms of both clinical symptoms and underlying genetic cause. Within the mitochondrial group, those patients with only clinically apparent muscle involvement had lower sNFL levels than patients with additional non-muscle symptoms. Thus, patients with a single deletion in mtDNA and manifesting CPEO only, had significantly lower sNFL levels than patients with CPEO + and a single or multiple deletions (due to a mutation in *POLG*) in mtDNA in muscle (Table 1 and Supplementary Figure 2; 11.3 ± 2 vs. 24.6 ± 5.3; mean ± SD; *p* < 0.05). The greatest range in sNFL levels occurred in the group of patients with a mutation in mtDNA, where the levels ranged from 11.9 (patient 17 with a heteroplasmic m.8344A > G mutation in tRNA lysine) to 1,250 pg/ml (patient 18, with a homoplasmic m.4290T > C mutation in tRNA Ile) (Table 1 and Supplementary Figure 2). Four patients from the mitochondrial group harbored a heteroplasmic m.3243A > G mutation in tRNA leucine 1 (the so-called MELAS mutation, Table 1). Although this group is small, the sNFL levels in these 4 patients showed no correlation with the mutation load in either blood or urine (Pearson correlation test, *p* = 0.7 and 0.6, respectively). Interestingly, the two patients with less severe neuronal involvement (P11 and P14 with polyneuropathy only) had similar sNFL levels 13.7 and 12.7 pg/ml), despite the difference in the underlying genetic cause. P11 harbors a heterozygous mutation in the nuclear gene *POLG*, whilst P14 harbors a m.9185T > C in the mitochondrial gene *MT-ATP6* which encodes a subunit of complex V.

## Discussion

This study demonstrates that sNfL levels can be used as a sensitive biomarker of ongoing neuronal damage in primary myopathies. As proof of concept, sNFL levels were significantly elevated in patients suffering from myopathies in which CNS-manifestations have been established, either as a clinically apparent symptom of the underlying genetic defect or by auxiliary clinical investigations (MRI, neurophysiology). Thus, we found sNfL to be high in patients with DM1, PROMM and mitochondrial disease, but also—even though to a lesser degree—in patients with FSHD. Our concept of sNfL as a biomarker for nervous system involvement in primary myopathies is further validated by the lack of sNfL elevations in patients from the disease control group (myopathies due to mutations in genes exclusively expressed in muscle tissue).

The severity of neuronal damage appeared to be loosely correlated with the degree of sNfL elevation: disease groups with clinically common and more severe CNS-symptoms (DM1, mitochondrial myopathy) showed on average higher median sNfL levels than disorders in which signs of CNS-involvement are mild and less common (PROMM/FSHD).

Within the Mito-group, patients with a single deletion in mtDNA and only CPEO had on average, a significantly lower sNfL level than patients with CPEO plus (e.g., P6 with ataxia and P9 with sensorineural hearing loss and cognitive impairment). In addition, the highest sNfL levels were observed in patients with the most severe involvement, even when the underlying mutation resides in the same mtDNA gene. Thus, in the two patients with a homoplasmic mutation in the mtDNA gene *MT-ATP6* (P14 and P15), the highest sNFL levels were detected in P15, with ataxia, dysarthria, epilepsy, optic atrophy, cognitive impairment and polyneuropathy as compared to P14 with polyneuropathy. The highest sNfL levels were observed in patients who experienced symptoms such as epilepsy, ataxia or stroke-like episodes (Table 1: P15, P16, P18, P19, P20). Patient 18 (described in Limongelli et al., 2004) had the highest sNFL levels of all of the mitochondrial patients (1,250 pg/ml), and she has the most severe neurological involvement of the group with Leigh disease with onset at the age of 16 years and necrotizing encephalopathy of basal ganglia and brain stem.

One of the greatest problems associated with reaching a prognosis in individuals who harbor a mutation in mtDNA is that these mutations are often heteroplasmic, i.e., both mutant and wild type mtDNA are present in an affected individual, and the mutation load may vary from tissue to tissue. The mutation level in urinary epithelial cells can be a useful predictor of the mutation load in muscle (Whittaker et al., 2009), but the extent to which the level in this tissue mirrors that in nervous tissue is unknown. Three of our four patients with the m.3243A > G mutation (tRNA leucine 1) harbor similar mutation loads in urinary epithelial cells, but show different degrees of neuronal involvement which suggests that the sNFL levels may be a better indicator of neuronal involvement than the degree of heteroplasmy in urinary epithelial cells. Thus, regular measurement of sNFL levels may provide an important prognostic tool for treatment of these patients. Indeed, higher sNfL concentrations in patients with MELAS-syndrome during acute stroke-like episodes than in the attack-free interval suggest the applicability of sNfL for the assessment of neuronal damage in mitochondrial diseases (Zheng et al., 2021). Also in our cohort, P20 had only manifested sensorineural hearing loss until the age of 61, when she developed stroke-like episodes with a very high sNFL level of 70.4 pg/ml.

Interestingly, only patients with DM I and DM2/PROMM showed a significant correlation of sNfL with age (Supplementary Figures 1A,B) within their cohort. This suggests that additional neuronal damage, above that which occurs with normal aging, accumulates in the myotonic dystrophies. Thus, our observations could support the concept of myotonic dystrophies as progeroid disorders with premature aging, as suggested previously (Meinke et al., 2018).

Regarding repeat length, sNfL correlated well to repeat length for the DM I patients, which is supported by previous observations of a negative correlation between brain volume on neuroimaging and CTG repeat length (van der Plas et al., 2019). However, one patient (P7) presented sNfL greatly above the range of all other DM I patients; this patient, as mentioned above, also exhibited clinical symptoms which indicated additional neuronal involvement separate from DM I.

In contrast, no correlation could be demonstrated between sNfL and repeat length in the DM2/PROMM patients. This is not surprising, however, considering the lack of a correlation between repeat length and the severity of the myopathy in these patients (Schoser, 2006).

Interestingly, even patients with FSHD demonstrated elevated sNfL compared to age matched controls, although CNS-involvement is not generally regarded to be clinically relevant in FSHD. However, neurophysiological (Stübgen, 2007) and cognitive (Sistiaga et al., 2009) assessment revealed alterations of CNS function in up to 70% of patients with FSHD.

Owing to the rarity of the myopathies examined in this study, the size of the disease groups was small, but despite this drawback, the estimated differences in sNfL levels were very high. Other confounding variables affecting NfL concentrations, such as a subclinical age-dependent vascular encephalopathy (Thebault et al., 2020), were accounted for by comparison with NfL values in a large group of healthy controls of a similar age range. Thus, NfL elevations due to an age-dependent encephalopathy in the patient groups would be matched in the age-adjusted controls. Other concomitant CNS-disease which might influence NfL levels, was excluded on clinical grounds.

We propose from this pilot study that sNfL could be used as simple and non-invasive biomarker to detect and monitor neuronal damage in myopathies, even in those without clinically evident CNS-involvement. sNfL might also be helpful to assess treatment effects in view of upcoming new gene therapies for myopathies with nervous system involvement, such as the dystrophinopathies, the myotonic dystrophies or mitochondrial disorders. Larger longitudinal studies comparing sNfL levels with imaging or neuropsychological course parameters, are required to elucidate the value of sNfL as an outcome parameter in clinical practice.

## Data Availability Statement

The original contributions presented in the study are included in the article/Supplementary Material, further inquiries can be directed to the corresponding author/s.

## Ethics Statement

The studies involving human participants were reviewed and approved by the Local Ethics committee of the Technische Universität Dresden (ID: EK394102018). The patients/participants provided their written informed consent to participate in this study.

## Author Contributions

AS, JS, and SJ designed the concept of the study. AS, JS, JK, PB, SJ, EW, TZ, and KA contributed to data acquisition and analysis. KA and TZ developed ethics protocol. AS, JS, SJ, and PB drafted the manuscript and figures. All authors contributed to the article and approved the submitted version.

## Conflict of Interest

The authors declare that the research was conducted in the absence of any commercial or financial relationships that could be construed as a potential conflict of interest.

## Publisher’s Note

All claims expressed in this article are solely those of the authors and do not necessarily represent those of their affiliated organizations, or those of the publisher, the editors and the reviewers. Any product that may be evaluated in this article, or claim that may be made by its manufacturer, is not guaranteed or endorsed by the publisher.
