# Supplementary material for: Serum Neurofilament Light Chain: A Marker of Nervous System Damage in Myopathies
Source: Front Neurosci. 2021 Dec 17;15:791670. doi: 10.3389/fnins.2021.791670 (PMC8718922; doi:10.3389/fnins.2021.791670)
Supplement: Supplementary file 1 [file Image_1.pdf]

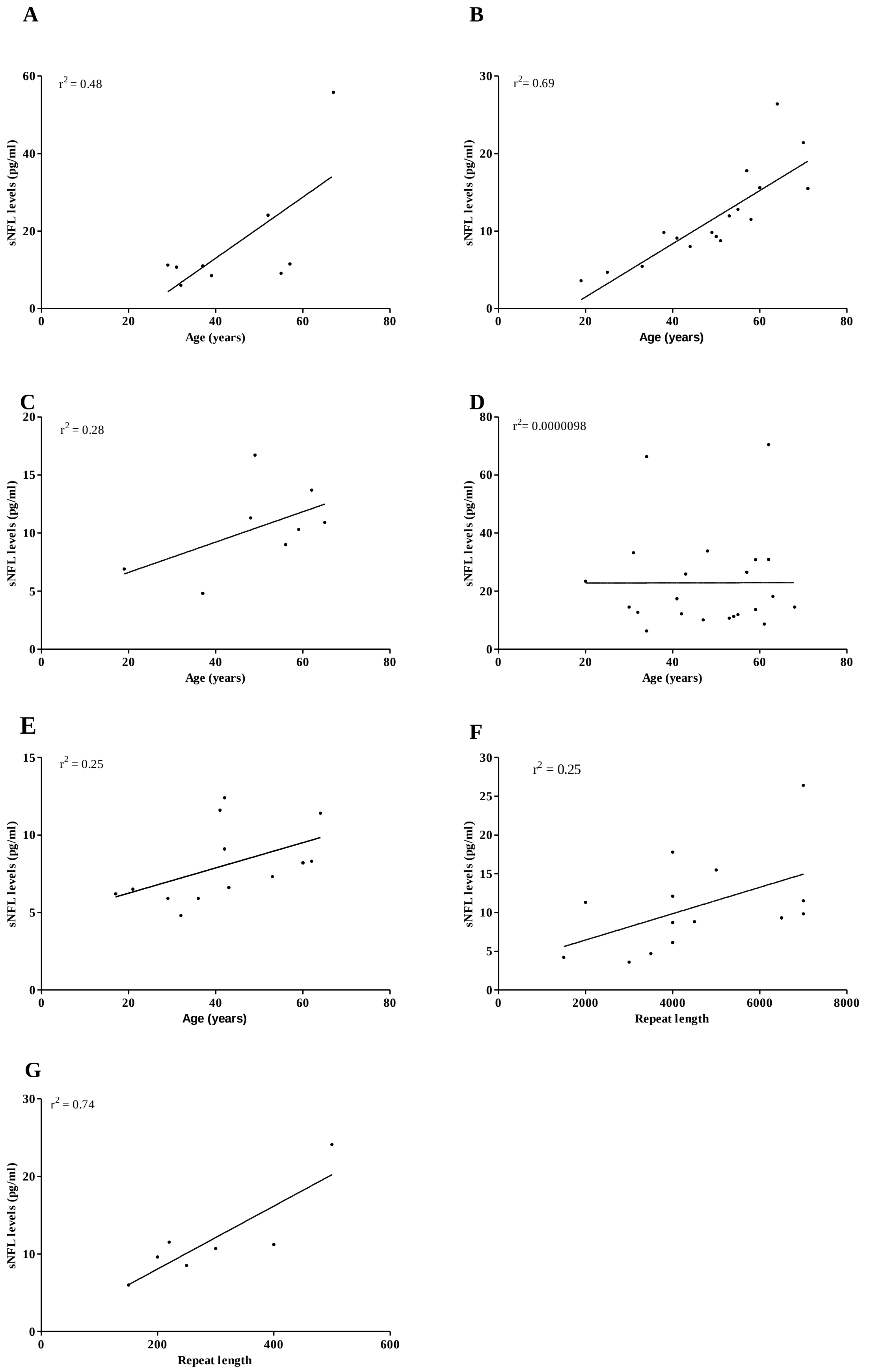

Supplementary Figure 1.

The sNFL levels are correlated with age in some patient cohorts, but not in others. sNFL levels as a function of age in A) the DM I patient cohort; B) the DM2/PROMM patient group; C) the FSHD group; D) the mitochondrial disease group and E) the muscle disease control group.

F) sNFL levels according to repeat length in DM2/PROMM patients, and G) sNFL levels according to repeat length in DM I patients.

A linear regression line was fitted to the data using GraphPad Prism 5, and the r^2 value is shown on each graph.
The Pearson correlation co-efficient was calculated for each data set. There was a significant correlation between age and sNFL levels in the DM I patients ($r = 0.69$, $p= 0.04$) and the DM2/PROMM patients ($r = 0.82$, $p<0.0001$). No significant correlation was found between age and sNFL levels in the FSHD group ($r = 0.53$; $p = 0.17$), the mitochondrial group ($r = 0.0009$; $p = 0.997$), or the disease control group ($r = 0.5$; $p = 0.08$).
No significant correlation was found between sNFL levels and repeat length in the DM2/PROMM patients ($r = 0.5$; $p = 0.07$), but a significant correlation was found between repeat length and sNFL levels in the DM I group of patients ($r = 0.86$; $p = 0.014$*). If P7 (outlier value, see text) is included, the correlation is lost because of the extent of the deflection and the low number patients ($r = 0.29$; $p = 0.53$).
