# Supplementary material for: Serum Neurofilament Light Chain: A Marker of Nervous System Damage in Myopathies
Source: Front Neurosci. 2021 Dec 17;15:791670. doi: 10.3389/fnins.2021.791670 (PMC8718922; doi:10.3389/fnins.2021.791670)
Supplement: Supplementary file 2 [file Image_2.pdf]

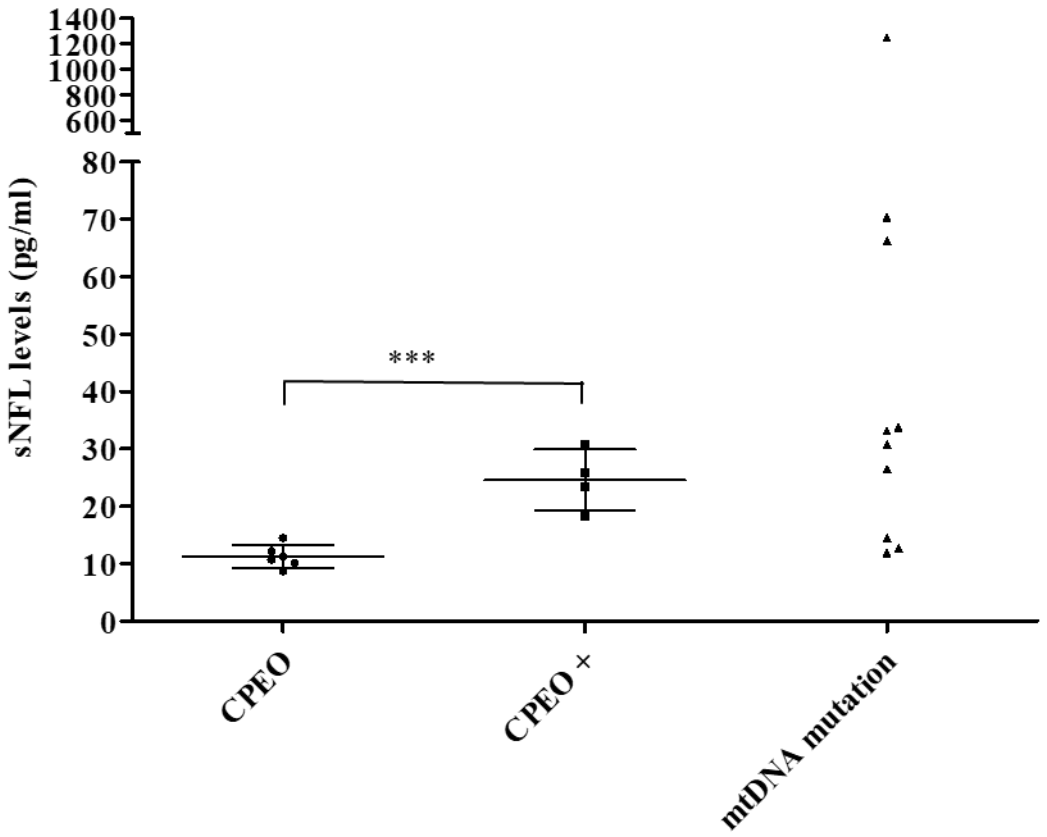

Supplementary Figure 2.
Differences in sNFL levels between selected groups of patients from the mitochondrial disease patient cohort. sNFL levels were measured in patients with CPEO associated with a single deletion in mtDNA in muscle, $CPEO^+$ patients with additional clinical features, associated with a single deletion in mtDNA, or in one case, multiple deletions in mtDNA in muscle and in patients who harbour a pathogenic sequence change in mtDNA. The values shown are mean ± SD. Statistical tests were performed using the unpaired t-test (***p <0.005).
